# Agitated Honeybees Exhibit Pessimistic Cognitive Biases

**DOI:** 10.1016/j.cub.2011.05.017

**Published:** 2011-06-21

**Authors:** Melissa Bateson, Suzanne Desire, Sarah E. Gartside, Geraldine A. Wright

**Affiliations:** 1 Centre for Behaviour and Evolution, Institute of Neuroscience, Newcastle University, Framlington Place, Newcastle upon Tyne NE2 4HH, UK

## Abstract

Whether animals experience human-like emotions is controversial and of immense societal concern [1–3]. Because animals cannot provide subjective reports of how they feel, emotional state can only be inferred using physiological, cognitive, and behavioral measures [4–8]. In humans, negative feelings are reliably correlated with pessimistic cognitive biases, defined as the increased expectation of bad outcomes [9–11]. Recently, mammals [12–16] and birds [17–20] with poor welfare have also been found to display pessimistic-like decision making, but cognitive biases have not thus far been explored in invertebrates. Here, we ask whether honeybees display a pessimistic cognitive bias when they are subjected to an anxiety-like state induced by vigorous shaking designed to simulate a predatory attack. We show for the first time that agitated bees are more likely to classify ambiguous stimuli as predicting punishment. Shaken bees also have lower levels of hemolymph dopamine, octopamine, and serotonin. In demonstrating state-dependent modulation of categorization in bees, and thereby a cognitive component of emotion, we show that the bees' response to a negatively valenced event has more in common with that of vertebrates than previously thought. This finding reinforces the use of cognitive bias as a measure of negative emotional states across species and suggests that honeybees could be regarded as exhibiting emotions.

**Video Abstract:**

## Highlights

► Agitated honeybees display an increased expectation of bad outcomes ► Hemolymph levels of dopamine, octopamine, and serotonin are reduced in agitated bees ► Honeybees exhibit a vertebrate-like emotional state

## Results and Discussion

Identifying the best objective measures of negative affect (i.e., emotion) in animals is currently the focus of intense debate [2, 4, 21, 22]. One approach that has recently received considerable attention is the measurement of biases in information processing that are typical of negative affective states—so-called “cognitive biases” [9]. Specifically, negative affective states such as anxiety are associated with increased expectation of punishment, greater attention to potential threats, and a tendency to interpret ambiguous stimuli as if they were threats (i.e., a “glass-half-empty” or pessimistic bias) [10, 11].

**Figure 1:**
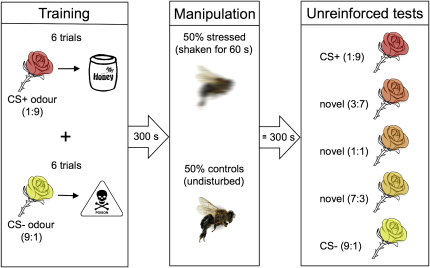
Protocol for Cognitive Bias Experiment with Olfactory Conditioning of Honeybees Honeybees were trained for six trials with each stimulus (CS) in a pseudorandomized sequence. The CS+ odor was a ratio of 1 part 1-hexanol to 9 parts 2-octanone; the CS− was a 9:1 ratio of the same two odors. After conditioning, bees were placed either in a group that was exposed to 60 s of shaking or in a control group. All bees began the testing session within 300 s of the manipulation. They were tested with each CS and three novel, intermediate ratios of the same two odors. All test trials were unreinforced, and the order of test odors was randomized across subjects.

We measured cognitive biases in honeybees subjected to a manipulation designed to induce an anxiety-like state using a similar approach to that adopted in studies of vertebrates [15, 17, 23]. Prior to any affective manipulation, subjects are required to learn that one stimulus (CS+) predicts reward, whereas another in the same sensory dimension (CS−) predicts punishment (or a reward of less value). Following a manipulation of state, the subjects' judgment is probed by testing their classification of novel stimuli with sensory properties intermediate between the two trained stimuli. A pessimistic cognitive bias is manifested in an increased tendency of subjects to classify stimuli as likely to predict punishment (or a reward of less value). We were able to use the same approach to test for cognitive biases in honeybees because bees are capable of associative learning and can base judgments about novel stimuli on previous experiences [24–27]. Using an olfactory learning protocol for conditioned proboscis extension, we trained honeybees to extend their mouthparts to a two-component odor mixture (CS+) predicting a reward (e.g., 1.00 or 2.00 M sucrose) and to withhold their mouthparts from another mixture (CS−) predicting either punishment or a less valuable reward (e.g., 0.01 M quinine solution or 0.3 M sucrose; Figure 1). The experiment comprised three conditions differing in the pairs of rewards and punishers used, to allow us to determine to what extent any differences in behavior were explained by the salience of the unconditioned stimuli (USs) used.

**Figure 2:**
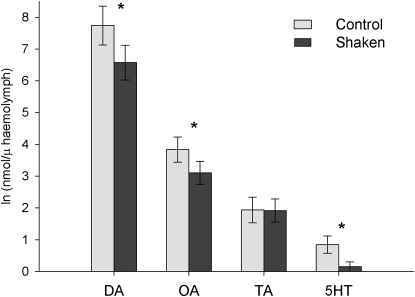
Vigorous Shaking for 60 Seconds on a Vortecizer Reduced the Levels of Biogenic Monoamines in Honeybee Hemolymph around 300 Seconds Later Dopamine (DA, F_1,24_ = 7.79, p < 0.011), octopamine (OA, F_(1,24)_ = 5.16, p < 0.034), and serotonin (5HT, F_1,24_ = 8.84, p < 0.007) all decreased, but the level of tyramine (TA, F_1,24_ = 0.041, p = 0.841) did not change. n_control_ = 12, n_shaken_ = 13. Actual (untransformed) mean values are as follows: DA, $\bar{x}$ unstressed = 8.42 ± 3.05 μM, stressed = 4.30 ± 1.75 μM; OA, $\bar{x}$ unstressed = 87.9 ± 25.3 nM, stressed = 63.4 ± 24.2 nM; 5HT, $\bar{x}$ unstressed = 2.38 + 1.02 nM, stressed = 0.781 ± 0.522 nM; TA, $\bar{x}$ unstressed = 14.4 ± 4.58 nM, stressed = 12.8 ± 4.07 nM. Error bars represent ± standard error of the mean (SEM).

Immediately after training, half of the honeybees were subjected to vigorous shaking for 60 s to simulate the state produced by a predatory attack on a concealed colony. Physical agitation is likely to be a good predictor of imminent attack in honeybees because brood predators and honey thieves such as the honey badger (*Mellivora capensis*) have been observed to use their accomplished digging skills to break into beehives [28]. To confirm that our shaking manipulation produced a physiological change, we used a different group of bees to measure changes in biogenic monoamine levels previously shown to be affected by shaking, spinning, or agitating [29, 30]. We found that 60 s of shaking significantly reduced constitutive levels of octopamine, dopamine, and serotonin in honeybee hemolymph at a time point following shaking that corresponded to when the cognitive tests reported below were performed (Figure 2).

**Figure 3:**
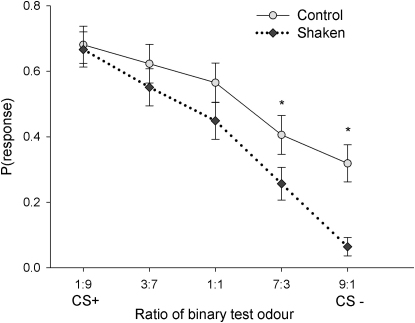
Shaken Honeybees Exhibit a Pessimistic Cognitive Bias When honeybees were subjected to shaking and then tested with the CS+, the CS−, and three novel odors, the slope of the gradient of the line indicating the proportion of bees that extended their proboscis [i.e., P(response)] became steeper (shaking × test odor interaction: χ_1_^2^ = 8.08, p = 0.005). The bees were significantly less likely to respond to the CS− and its adjacent novel odor (^∗^p < 0.05). The data represent the pooled responses from all three unconditioned stimulus (US) treatments reported in Figure S1. n_control_ = 69, n_shaken_ = 78; error bars represent ± 1 SEM.

We observed that shaken bees exhibited pessimistic judgment biases. Within 5 min of the shaking manipulation, all of the trained bees began a sequence of unreinforced test trials with five odor stimuli presented in a randomly chosen order for each bee: the CS+, the CS−, and three novel odors composed of ratios intermediate between the two learned mixtures. Shaken honeybees were more likely to withhold their mouthparts from the CS− and from the most similar novel odor (Figure 3; Table 1). This effect was independent of the pair of USs used (see Figure S1 available online; US × test odor × shaken interaction, logistic regression χ_2_^2^ = 0.24, p = 0.889), suggesting that the observed effect of shaking is robust and does not depend on which positive and negative USs are experienced during conditioning. Furthermore, the reduction in likelihood of responses seen in the shaken bees did not reflect a general effect of stress on olfactory sensory processing or motivation to respond because shaken bees' responses to the CS+ were unaffected (least-squares contrast, χ_1_^2^ = 0.14, p = 0.706). Therefore, our data show that shaken bees alter their classification of ambiguous test odors and particularly the CS−.

Our study differs from previous cognitive bias studies in showing that the greatest effect of the negative manipulation is on the response to the CS−, as opposed to just the novel, ambiguous stimuli. We were able to measure a reduction in responding to the CS− because a percentage of our subjects still responded as if it signaled the CS+ after conditioning. The fact that some bees still extended the proboscis to the CS− indicates that our conditioning task was difficult for the bees to perform [25]. Furthermore, cognitive bias has previously only been studied in vertebrate animals where the subjects experienced many conditioning trials over several days, whereas our bees were tested 10 min after receiving only 12 conditioning trials. The cognitive bias we observed indicates that the shaken bees have an increased expectation of punishment (i.e., receiving the US associated with the CS−). This could reflect a change in either the bees' perception of the probability of punishment or the impact of punishment [4].

Our results add an invertebrate animal to the growing list of vertebrates, including rats [12, 14, 15], sheep [13], dogs [16], starlings [17–19], and domestic chicks [20], that when subjected to various forms of negative, stressful manipulations exhibit pessimistic judgment biases. Our findings therefore strengthen the hypothesis that pessimistic judgment biases are likely to be a good measure of negative emotional states across species because they are tightly linked to the evolutionary function of these states [4, 9, 11, 31].

Previous research has established that honeybees, like vertebrates, possess the cognitive sophistication to generalize from one stimulus to another based on its consequences rather than its sensory properties [25, 27, 32]. Our data are the first to demonstrate that, as in human subjects, state alters a honeybee's judgments toward signals associated with potential threats to fitness. The physiological mechanisms that produce this change are poorly understood. In honeybees, octopamine is the local neurotransmitter that functions during reward learning [33], whereas dopamine mediates the ability to learn to associate odors with quinine punishment [34]. In insects, these monoamines are also constitutive hormones. Our data suggest that constitutive levels of octopamine, dopamine, or serotonin influence the way that the neural circuits involved in cognitive generalization function. In *Drosophila*, constitutive serotonin affects the expression of aggression in male flies. If flies are fed serotonin, they are more aggressive; flies depleted of constitutive serotonin still exhibit aggression, but they do so much less frequently, indicating that the neural circuits involved in this behavior still function [35]. If the insect brain is organized such that the same neural circuits are involved in both reward learning and punishment [36], fluctuations in hemolymph serotonin, octopamine, and dopamine caused by an acute stressor could affect the expression of olfactory memories by acting directly on the circuits encoding them and hence lead to the cognitive bias that we observe.

Using the best criteria currently agreed on for assessing animal emotions, i.e., a suite of changes in physiology, behavior, and especially cognitive biases [4–8], we have shown that agitated bees display a negative emotional state. Although our results do not allow us to make any claims about the presence of negative subjective feelings in honeybees, they call into question how we identify emotions in any nonhuman animal. It is logically inconsistent to claim that the presence of pessimistic cognitive biases should be taken as confirmation that dogs or rats are anxious but to deny the same conclusion in the case of honeybees.

## Experimental Procedures

### Subjects

Individual worker honeybees (*Apis mellifera carnica*) were collected from an outdoor colony maintained at Newcastle University, restrained in harnesses [25], fed to satiety with 1.0 M sucrose, and left for ∼24 hr.

### Behavioral Experiments

Honeybees were conditioned with two odors, each paired with a different outcome, presented in a pseudorandom sequence (ABBABAABABBA, where A = CS+ and B = CS−) with an intertrial interval of 5 min for a total of 12 trials using an established protocol for conditioned proboscis extension [24]. The odors, 1-hexanol and 2-octanone (99.8% purity, Sigma-Aldrich), were combined as a binary mixture in mineral oil and used as the conditioned stimuli and test odors in the following proportions: 1:9, 3:7, 1:1, 7:3, and 9:1. The overall concentration of the mixtures was 2.0 M in solution (methods described in [25]). The 1:9 odor mixture was always presented with a rewarding food solution, and the 9:1 mixture was always presented with a punishing or less rewarding solution; previous studies have demonstrated that both mixtures are learned equally well [26]. The two odors used in these blends were chosen because they are general odors that have similar perceptual properties (i.e., the honeybee antennal neurons exhibit the same concentration tuning) [37, 38]. We do not have any reasons a priori to expect that shaking-induced stress should bias the response of bees toward or away from either odor in this blend. We chose not to counterbalance the use of the ratios at CS+ or CS− to make the experimental protocol simpler to execute and, therefore, less prone to experimenter error.

Three combinations of reward and punishment were used during conditioning: (1) 1.0 M sucrose (CS+) versus 0.3 M sucrose (CS−), (2) 1.0 M sucrose (CS+) versus 0.01 M quinine (CS−), and (3) 2.0 M sucrose (CS+) versus 0.01 M quinine (CS−), with individual bees being assigned to one of these three US conditions. After conditioning, half of the conditioned honeybees (“shaken” group) were subjected to 60 s of vigorous shaking on a Vortex-T Genie 2 with a modified attachment (Scientific Industries) while the other half (“control” group) were left undisturbed. Within 5 min after shaking, both groups were tested with all five stimuli without reinforcement; the order of presentation was randomized across subjects. Bees were trained, manipulated, and tested in groups of ten animals, half of which were shaken and half of which acted as controls. Therefore, any effects of potential confounding variables, such as time or day, had an equal impact on both shaken animals and controls.

### Biogenic Monoamine Measurement

Using a 10 μl glass capillary tube, hemolymph was acquired from a hole pierced through the exoskeleton of the head capsule near to the median ocellus. The hemolymph was immediately placed into a microcentrifuge tube containing 20 μl of 0.1 M perchloric acid on ice. Composite samples were acquired from 5–15 bees to a volume of ∼20 μl. The sample was brought to a final volume of 100 μl with perchloric acid and centrifuged for 5 min at 13,000 rpm. The supernatant taken was taken off and frozen at −20°C. Subsamples of the hemolymph were diluted to a 1:4 concentration in the mobile phase prior to analysis. Biogenic amines were analyzed using high-performance liquid chromatography (HPLC) with electrochemical detection (Coulochem II, ESA) with a guard cell and a porous graphite “frit” flow cell both set at 660 mV. A C18 reverse-phase column (3 μm microsorb, 100 mm × 4.6 mm) was heated to 40°C. The mobile phase consisted of 50 mM citrate/acetate (pH 4.5), 20% acetonitrile, and 11 mM decanesulfonic acid using a method by Hardie and Hirsh [39] and flowed at a rate of 1 ml/min.

### Data Analysis

In the behavioral experiments, the measured response variable was whether a honeybee extended its proboscis in response to stimulation or not (a binary variable). Therefore, a repeated-measures, logistic regression analysis (SAS, PROC GENMOD) was used to analyze the data. The model fitted included test odor (continuous), shaking (two levels: shaken or control), US type (three levels corresponding to the three different US pairs described above), and their interactions as independent variables (Table 1). Because US type did not explain significant variation in the behavior of the bees, we pooled the data from the three US conditions for the purposes of presentation in Figure 3. One-tailed least-squares multiple comparisons (LSC) were conducted to make specific pairwise comparisons between the 1:9 odor mixture and the other test odors. For the biogenic amine analysis, data were natural log transformed prior to entry in a two-way multivariate analysis of variance with shaking (two levels: shaken or control) as the sole independent variable.

## Figures and Tables

**Table 1:** Logistic Regression for the Responses of Honeybees to the Five Test Odors

Variable	df	χ^2^	p Value
Test odor (continuous)	1	88.2	<0.001^∗^
Shaken (two levels)	1	1.76	0.185
US (three levels)	2	4.90	0.086
Test odor × shaken	1	8.08	0.005^∗^
Test odor × US	2	0.92	0.631
Shaken × US	2	0.66	0.717
Test odor × US × shaken	2	0.24	0.888

“Shaken” refers to whether or not the bees experienced the shaking treatment, and “US” refers to the reinforcer and punisher used during differential conditioning. ^∗^p < 0.05.

## Supplemental Information

Document S1. One Figure
