# Supplementary material for: Agitated Honeybees Exhibit Pessimistic Cognitive Biases
Source: Curr Biol. 2011 Jun 21;21(12):1070–3. doi: 10.1016/j.cub.2011.05.017 (PMC3158593; doi:10.1016/j.cub.2011.05.017)
Supplement: Document S1. One Figure [file mmc1.pdf]

Current Biology, Volume *21*

Supplemental Information

Agitated Honeybees Exhibit

Pessimistic Cognitive Biases

Melissa Bateson, Suzanne Desire, Sarah E. Gartside, and Geraldine A. Wright

Supplemental Inventory

1. Supplemental Figure

Figure S1, related to Figure 3

Figure S1. Related to Figure 3.
After being shaken, honeybees respond to the CS- and similar novel odors as if these odors are more likely to predict punishment. The strength of the reward and the punisher used during differential conditioning influenced the shape of the generalization gradient ($\chi_2^2 = 13.1$, $p = 0.001$) but did not affect the expression of the pessimistic cognitive bias ($\chi_2^2 = 0.24$, $p = 0.888$). Honeybees were differentially conditioned with one of three treatments: (a) the CS+ was associated with 1.0 M sucrose and the CS- was associated with 0.3 M sucrose, $n_{control} = 36$, $n_{shaken} = 38$; (b) the CS+ was associated with 1.0 M sucrose and the CS- was associated with 0.01 M quinine, $n_{control} = 20$, $n_{shaken} = 25$; (c) the CS+ was associated with 2.0 M sucrose and the CS- was associated with 0.01M quinine, $n_{control} = 13$, $n_{shaken} = 15$. Error bars represent $\pm$ 1 SEM.

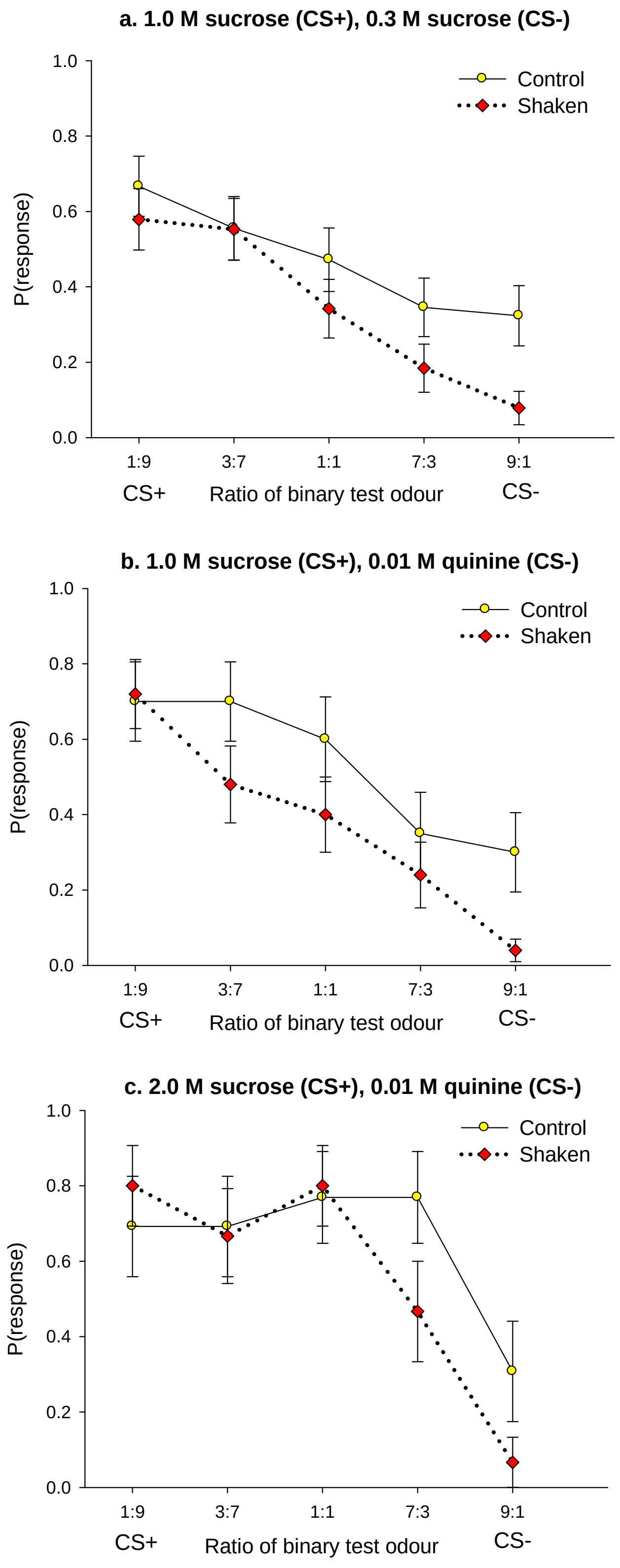
a. 1.0 M sucrose (CS+), 0.3 M sucrose (CS-)
P(response)
1.0
0.8
0.6
0.4
0.2
0.0
Control
Shaken
1:9
3:7
1:1
7:3
9:1
CS+
Ratio of binary test odour
CS-
b. 1.0 M sucrose (CS+), 0.01 M quinine (CS-)
P(response)
1.0
0.8
0.6
0.4
0.2
0.0
Control
Shaken
1:9
3:7
1:1
7:3
9:1
CS+
Ratio of binary test odour
CS-
c. 2.0 M sucrose (CS+), 0.01 M quinine (CS-)
P(response)
1.0
0.8
0.6
0.4
0.2
0.0
Control
Shaken
1:9
3:7
1:1
7:3
9:1
CS+
Ratio of binary test odour
CS-
